# Mitochondrial Control of Stem Cell State and Fate: Lessons From *Drosophila*

**DOI:** 10.3389/fcell.2021.606639

**Published:** 2021-05-03

**Authors:** Satish Kumar Tiwari, Sudip Mandal

**Affiliations:** ^1^Developmental Genetics Laboratory, Department of Biological Sciences, Indian Institute of Science Education and Research (IISER) Mohali, Mohali, India; ^2^Molecular Cell and Developmental Biology Laboratory, Department of Biological Sciences, Indian Institute of Science Education and Research (IISER) Mohali, Mohali, India

**Keywords:** *Drosophila*, metabolism, mitochondria, stem cell, regulation, maintenance, differentiation

## Abstract

Over the years, *Drosophila* has served as a wonderful genetically tractable model system to unravel various facets of tissue-resident stem cells in their microenvironment. Studies in different stem and progenitor cell types of *Drosophila* have led to the discovery of cell-intrinsic and extrinsic factors crucial for stem cell state and fate. Though initially touted as the ATP generating machines for carrying various cellular processes, it is now increasingly becoming clear that mitochondrial processes alone can override the cellular program of stem cells. The last few years have witnessed a surge in our understanding of mitochondria’s contribution to governing different stem cell properties in their subtissular niches in *Drosophila*. Through this review, we intend to sum up and highlight the outcome of these *in vivo* studies that implicate mitochondria as a central regulator of stem cell fate decisions; to find the commonalities and uniqueness associated with these regulatory mechanisms.

## Introduction

Adult stem cells are rare populations of undifferentiated cells that reside among the differentiated cells in fully developed tissues. During the postnatal life, these multipotent cells possess two defining characteristics: (a) the capacity to self-renew themselves and (b) differentiate into a limited number of mature cell types (Fuchs and Chen, 2012; Sánchez Alvarado and Yamanaka, 2014). Usually, the adult stem cells are maintained in a quiescent state (Cheung and Rando, 2013), and they proliferate and differentiate only upon activation (Post and Clevers, 2019). In doing so, they are dynamically involved in remodeling the tissue in response to turnover (Barker et al., 2010), damage (Goldman and Poss, 2020), and disease (Clevers and Watt, 2018; Naik et al., 2018). Therefore, the precise balance between adult stem cell self-renewal and differentiation is crucial for tissue growth and homeostasis.

Within a tissue, adult stem cells are located in a specialized microenvironment termed as the niche (Schofield, 1978; Kimble and White, 1981). Though the actual architecture of the niche and its components vary for different tissues, the niche typically has a spatial organization that provides anatomical and functional interactions critical for stem cell maintenance (Xie and Spradling, 1998), their proliferation (Kiger, 2001), and fate specification (Ferraro et al., 2010). Mutual and dynamic cell-cell interaction between niche and stem cells that involve secreted factors and signaling molecules elaborated or induced by niche cells (Huelsken et al., 2001; Chacon-Martinez et al., 2018), niche ECM proteins (Fuchs et al., 2000), and the physical forces attributed by niche’s mechanical scaffold (Kahn et al., 2009) and contribute significantly in maintaining the stem cell identity (Morrison and Spradling, 2008; Rezza et al., 2014). Apart from these local signals, increasing evidence highlights a pivotal role for distinct metabolic, systemic, and environmental signals in stem cell physiology and lineage specification (Drummond-Barbosa, 2008, 2019; Murphy, 2008; Shyh-Chang et al., 2013; Speder and Brand, 2018). Importantly, it is gradually becoming apparent from the recent findings that metabolites (Harvey et al., 2019) and nutrients (Ochocki and Simon, 2013) modulate stem cell fate by actively participating in controlling their intracellular signaling and enzymatic activities (Shyh-Chang and Ng, 2017). The role of metabolic intermediates in changing the epigenetic landscape of the stem cells and their progenies in terms of histone modifications (Yucel et al., 2019) and DNA methylation (DiTroia et al., 2019) is also much appreciated.

Mitochondria, classically known as “powerhouses” of a cell, are responsible for ATP production through oxidative phosphorylation (Oxphos) and sustained electron transport chain (ETC) activity. Besides their fundamental role in energy harvesting, mitochondria compartmentalize several metabolic pathways, such as the TCA cycle, fatty acid β-oxidation (FAO), and steroid metabolism (Newmeyer and Ferguson-Miller, 2003; McBride et al., 2006; Nunnari and Suomalainen, 2012). Functioning as a metabolic hub, mitochondria also play a critical role in integrating cell-intrinsic and extrinsic signals to regulate diverse processes that include calcium homeostasis (Vasington and Murphy, 1962), inflammation (Pearce and Pearce, 2013) and apoptosis (Green and Reed, 1998). Moreover, reactive oxygen species (ROS) generated as a byproduct of Oxphos, and the terminal and intermediate metabolites produced within the mitochondria can also act as retrograde signals to dictate gene expression, post-translational protein modifications, and bring about epigenetic modifications (Liu and Butow, 2006; Murphy, 2008; Tait and Green, 2012). Historically, relatively less attention was given to understand the role of mitochondria in stem cell biology, presumably because stem cells generally possess non-fused, spherical (immature form) mitochondria with poorly developed cristae (Lisowski et al., 2018). Therefore, it **is generally believed** that the stem cells rely more on glycolysis to generate energy for their proliferation. However, several studies in the last decade have unveiled the importance of mitochondria in controlling stem cell behavior, including their decisions to self-renew or differentiate (Mandal et al., 2011; Wanet et al., 2015; Bahat and Gross, 2019; Hinge et al., 2020).

The fruit fly, *Drosophila melanogaster*, is a versatile model organism used extensively in biomedical research to garner valuable information about regulatory pathways that facilitate our understanding of parallel pathways in humans. With the availability of sophisticated genetic tools, an extensive collection of mutants, and relatively easy accessibility to tissues, flies have provided a fantastic opportunity for *in vivo* analyses of adult stem cells (Yamashita et al., 2005; Jennings, 2011; Losick et al., 2011). To date, several stem cell populations have been identified in adult flies. While the Germline Stem Cells (GSCs), the Follicle Stem Cells (FSCs), the Escort Stem Cells (ESCs), and the Cyst Stem Cells (CyScs) are associated with the adult gonads (Margolis and Spradling, 1995), the adult gut harbors the Intestinal Stem Cells (ISCs; Micchelli and Perrimon, 2005; Ohlstein and Spradling, 2005; Takashima et al., 2008). Moreover, the presence of Muscle Stem Cells (MSCs; Chaturvedi et al., 2017) and Renal Stem Cells (RSCs; Singh et al., 2007) has been reported in the adult muscles and Malpighian tubules, respectively. Besides these, several stem cell populations that include Hematopoietic Stem Cells (HSCs; Dey et al., 2016) and Neural Stem Cells (NSCs; Truman and Bate, 1988; Bahat and Gross, 2019) are present as transient populations of stem cells during post-embryonic development. Studies of these different populations of stem cells have provided a high-resolution picture of the molecular mechanisms and cellular properties of stem cells that are conserved in mammals.

This review sets out to provide a comprehensive account of the recent advances in our understanding of mitochondria’s role in regulating GSCs, ISCs, NSCs, and hemocyte progenitors in *Drosophila*. In this context, it is essential to note that the role of mitochondria in HSCs, MSCs, and RSCs is yet to be implicated. Given that most of the analyses have been done *in vivo*, the findings are much more physiologically relevant. They provide relatively more accurate information about the importance of mitochondrial function in stem cell biology. Finally, we would highlight the mitochondrial regulatory mechanisms that are remarkably conserved across the different stem cell types, in contrast to those unique for a specific kind of stem cell.

## Germline Stem Cells in *Drosophila*

Production of gametes (sperms and egg) in adult individuals relies upon a robust stem cell system capable of balancing self-renewal with differentiation. Irrespective of its nature, gametes produced from the GSCs hold the key to the perpetuation of a species. *Drosophila* GSCs are established during development and function throughout the reproductive life to produce gametes (McKearin and Spradling, 1990). Like all other organisms, the *Drosophila* GSCs reside in a defined anatomical niche that provides necessary signals to maintain the precise balance between GSC self-renewal and differentiation (Fuller and Spradling, 2007). In addition, the GSCs and their developing progenies also sense and respond to a plethora of systemic factors governed by diverse physiological inputs that include diet intake, the organism’s metabolic status, and other environmental factors (Drummond-Barbosa and Spradling, 2001; Ting, 2013; Drummond-Barbosa, 2019; Kahney et al., 2019). Given the intricate nature of integrating local and systemic signaling pathways to modulate the state and fate of GSCs, the germline has emerged as one of the best models for studying adult stem cell biology *in vivo* (Greenspan et al., 2015). Our understanding of the *Drosophila* GSCs has identified a wide range of mechanisms by which metabolic status in general and mitochondrial function, in particular, dictates GSC activities.

## Mitochondrial Regulation of Male GSCs in *Drosophila*

**Figure 1:**
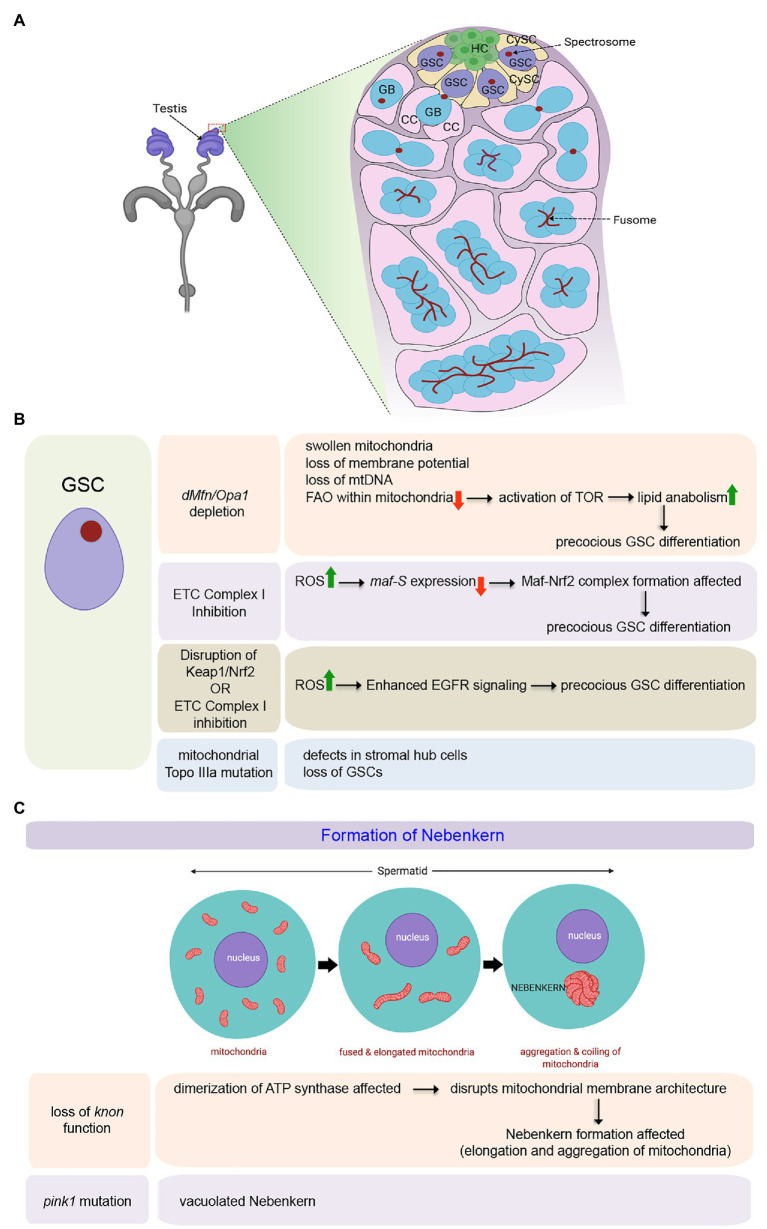
Role of mitochondria in maintenance and differentiation of male Germline Stem Cells (GSCs) in *Drosophila*. **(A)** Schematic representation of the tip of the adult testis showing the anatomical position of male GSCs and other associated cell types. The expanded forms of the acronyms used are provided in the text. **(B)** Table showing the diverse outcomes of manipulating mitochondrial structure and function in the male GSCs. **(C)** Schematic representation for mitochondrial fusion and aggregation to form the Nebenkern in the spermatids. Loss of *knon* and *pink1* function affects Nebenkern formation.

The *Drosophila* testis (Figure 1A) harbors around 9–11 GSCs at its apical tip adjacent to a cluster of non-diving somatic cells known as the hub (HC) that serves as a primary cellular component of the male GSC niche (de Cuevas and Matunis, 2011). GSCs divide asymmetrically to produce two daughter cells. One daughter cell that remains close to the hub retains the stem cell identity. Simultaneously, the other that is displaced away from the hub (referred to as a goniablast, GB) initiates the differentiation (Figure 1A). The goniablast undergoes four rounds of mitotic divisions with incomplete cytokinesis to give rise to a cyst of 16 interconnected spermatogonia. In addition, each GSC remains surrounded by a pair of CySCs (Figure 1A), which divides and differentiates into cyst cells (CC), that form a protective layer around the developing spermatogonia. Spermatogonia differentiate into spermatocytes, which by meiotic division give rise to spermatids that eventually mature to form sperms (Papagiannouli, 2014).

Mitochondria are highly dynamic organelles capable of changing their shape continuously undergoing fission or fusion. Significantly, change in their morphology is closely related to their functionality (Westermann, 2010). The two genes, *Drosophila* homolog of *Mitofusin* (known as *dMfn* or *Marf*) and *Optic Atrophy 1* (*Opa1*), responsible for the fusion of the outer and inner mitochondrial membranes, respectively, have been shown to play a pivotal role in maintaining the male GSCs (Sênos Demarco et al., 2019). It has been demonstrated that a block in mitochondrial fusion, either by *dMfn* or *Opa1* depletion, leads to a gradual loss of GSCs over time as they undergo precocious differentiation (Figure 1B). In the absence of fusion, mitochondria within the GSCs become swollen with aberrant ultrastructures, including collapsed cristae. Other hallmarks of these aberrant mitochondria include loss of membrane potential, loss of mitochondrial DNA (mtDNA), and decreased distribution of mitochondrial contents without significant upregulation in the ROS levels. Mechanistically, the inability of GSCs to self-renew and its loss due to differentiation in the absence of mitochondrial fusion is instigated by lipid anabolism and increased lipid accumulation in these cells. In the absence of an active and fused mitochondrial network, FAO within the mitochondria gets significantly affected, resulting in an increase in the level of fatty acids within the GSCs. Increased fatty acid levels, in turn, activates TOR that stimulates SREBP-mediated lipid anabolism, leading to further accumulation of lipids through a positive feedback mechanism. Under these conditions, male GSCs fail to maintain and eventually differentiate (Figure 1B; Sênos Demarco et al., 2019). In this context, it is essential to note that the independent knockdown of FAO enzymes, either by genetic or by pharmacological means, also leads to a similar phenotype, as seen for blocking mitochondrial fusion in the GSCs (Sênos Demarco et al., 2019). The involvement of mitochondrial FAO in the maturation of sperm has also been evidenced in flies mutant for the gene *scully* (Barbas et al., 1998), which codes for the mitochondrial 3-hydroxyacyl-CoA dehydrogenase responsible for the conversion of hydroxyl-acyl-CoA to ketoacyl-CoA during FAO. *scully* mutant testes are reduced in size and lack maturing sperm. The mutant spermatocytes are characterized by the accumulation of cytoplasmic lipid inclusions and scarce and aberrant mitochondria. Thus mitochondrial fusion, which in turn supports increased FAO, is essential for the maintenance of the male GSCs.

The role of mitochondrial fission, on the other hand, has not been implicated in the maintenance of GSCs in adult flies (Senos Demarco and Jones, 2019). Mutant clones of the pro-fission gene, *Dynamin-related protein* (*Drp1*), have otherwise normal GSC numbers and function in adults. However, Drp1 does play a role in the maintenance of early germ cells in the larval testis. A block in mitochondrial fission by *Drp1* depletion results in elevated ROS levels in the germ cells that led to activation of the EGFR pathway in the somatic cyst cells (Senos Demarco and Jones, 2019). This, in turn, induces a loss in GSC number and spermatogonia due to premature differentiation.

Inhibition of ETC complex I component ND75 (*Drosophila* homolog of human mitochondrial Complex I subunit NDUFS) also leads to precocious GSC differentiation by increased ROS levels in the *Drosophila* testis (Figure 1B; Tan et al., 2018). Gene expression profiling of the testes expressing *ND75RNAi* under the control of *nos*-*Gal4*, which is expressed in early-stage germ cells, identified Maf-S [a family member of basic region leucine zipper (bZIP)-type transcription factor Maf] as one of the effector molecules responsible for causing high ROS mediated early differentiation of GSCs. *maf-S* is transcriptionally downregulated by oxidative stress in the *ND75* knocked down testis (Figure 1B). Independently, it has been demonstrated that in wild-type flies while knockdown of *maf-S* leads to GSC differentiation, *maf-S* over-expression leads to a tumorous overgrowth of GSC-like cells. The precocious differentiation of GSCs as observed upon oxidative stress can be suppressed by ectopic expression of Maf-S, thereby demonstrating that Maf-S acts as a downstream effector of ROS signaling. Mechanistically, Maf-S interacts genetically with the Keap1-Nrf2 redox management system to regulate ROS-associated GSC behavior in the *Drosophila* testis (Tan et al., 2018). The Kelch-like ECH-associated protein 1 (Keap1)-NF-E2-related factor 2 (Nrf2) redox management system is a crucial regulator of cellular redox homeostasis (Suzuki et al., 2019). Oxidative stress leads to the disruption of Keap1-mediated proteasomal degradation of Nrf2. As a result, the stable Nrf2 translocates into the nucleus to hetero-dimerize with the Maf proteins. Together, they transcriptionally activate genes associated with detoxification that include *thioredoxin reductase* and *glutathione reductase*. In the case of GSCs with attenuated Complex I activity, due to a reduction in *maf-s* expression, the formation of the Maf-Nrf2 complex gets affected. In turn, this might disrupt the expression of some detoxification genes, allowing the GSCs to differentiate. Though, the target genes that lead to precocious differentiation are yet to be known (Tan et al., 2018), these results further endorse the importance of fused mitochondria and Oxphos in GSC maintenance. A separate study has also demonstrated that reducing ROS levels either by inhibiting the activity of Keap1 or upon antioxidant treatment promotes the overgrowth of GSC-like cells (Tan et al., 2017). Whereas, reduction in GSC number due to precocious differentiation is associated with elevated levels of ROS induced by alteration in Keap1/Nrf2 activity. Notably, this ROS mediated GSC differentiation, observed either upon disrupting the Keap1/Nrf2 activity or upon attenuating ETC Complex I activity (Figure 1B), is an outcome of enhanced EGFR signaling as the expressions of the EGFR ligand *spitz* gets increased. In consistence, significantly enhanced p-Erk1/2 expression is detected in CySCs and cyst cells that provide the necessary cues for differentiation (Figure 1B; Tan et al., 2017).

Significant changes in the mitochondrial network characterize the development of spermatids from GSC (Tokuyasu, 1975; Sênos Demarco et al., 2019). The network of mitochondria gets reticular with each developmental stage. Following meiosis, they dramatically fuse to form a giant layered spherical structure in the spermatids termed as the Nebenkern (Figure 1C; Hales and Fuller, 1997). Apart from its distinct physical appearance, the Nebenkern has been reported to have an unconventionally large paralog of ATP synthase subunit d. The gene *knotted onions* (*knon*) encodes a testis-specific paralog of ATP synthase subunit d, essential for Nebenkern’s formation and subsequent dynamics (Sawyer et al., 2017). Knon has been proposed to prevent the mitochondrial inner membrane’s sharp positive curvature within the Nebenkern by altering ATP synthase’s dimerization. As a result, during Nebenkern formation, the inner mitochondrial membrane shows very shallow curvature and stays closely apposed to the outer membrane. This unusual mitochondrial membrane configuration is critical for mitochondrial elongation. Loss of Knon function disrupts the typical mitochondrial membrane architecture within the Nebenkern that, in turn, causes aberrant mitochondrial elongation leading to male sterility (Figure 1C; Sawyer et al., 2017). Another study has also evidenced the association of other ATP synthase subunits with defects in germ cell maturation (Yu et al., 2019). For instance, knockdown of the ATP synthase β subunit in early germ cells hinders germ cell maturation without any apparent effect on GSCs and spermatogonia. The hub and cyst cells also remain unaffected. Other major ATP synthase subunits, upon their loss, also impede germ cell maturation (Yu et al., 2019).

Control of GSC activity in the adult testis by mitochondrial function has also been evidenced in a few more independent studies. For instance, 33% of males with defects in mitochondrial Topoisomerase IIIα (a member of the conserved Type IA subfamily of topoisomerases) are completely sterile (Wu et al., 2010). In contrast, the rest have lingering fertility, which gets lost in 6 days. Topo IIIa mutant males progressively lose GSCs with concomitant defects in stromal hub cells outlining the stem cell niche (Figure 1B). Furthermore, inhibition of *Drosophila pink1* leads to male sterility with defects in mitochondrial morphology and increased sensitivity to oxidative stress (Clark et al., 2006). In *pink1* mutant males, though the nuclei appear to be normal, spermatids have vacuolated Nebenkerns (Figure 1C). Interestingly, the expression of human *PINK1* in the *Drosophila* testes restores male fertility and normal Nebenkern morphology in *pink1* mutants. Furthermore, Pink1 works in conjunction with Parkin, as loss of their individual function phenocopies each other and the defects of *pink1* mutants can be rescued by overexpression of *parkin* (Clark et al., 2006).

## Mitochondrial Regulation of Female GSCs in *Drosophila*

**Figure 2:**
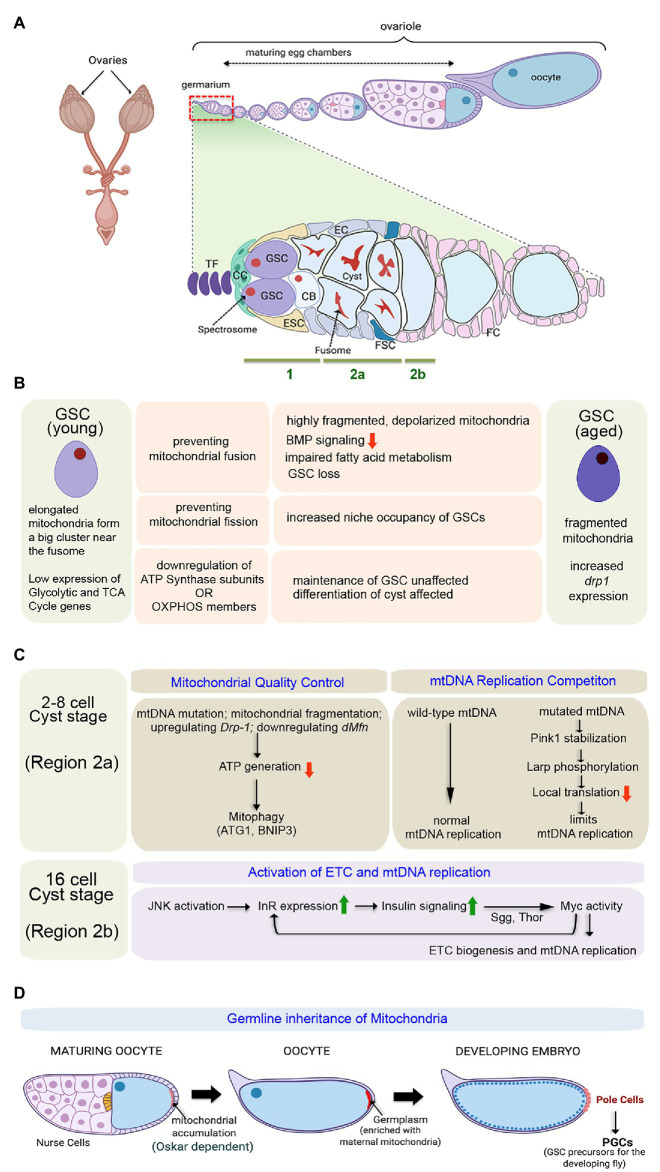
Importance of mitochondria in governing proliferation and differentiation of female GSCs in *Drosophila*. **(A)** Schematic representation of the adult ovary, a single ovariole showing the relative position of the germarium and the developing egg chambers, and a magnified view of the germarium displaying the anatomical position of the female GSCs, niche components, Escort Stem cells (ESCs), and Follicle Stem Cells (FSCs). The expanded forms of the acronyms used are provided in the text. **(B)** Table showing the diverse outcomes of manipulating mitochondrial structure and function in the young female GSCs. While young GSCs have elongated mitochondria, the aged ones are characterized by fragmented mitochondria. **(C)** Schematics of the pathways involved in mitochondrial quality control, mitochondrial DNA (mtDNA) replication competition, and activation of electron transport chain (ETC) and mtDNA replication in the developing cysts. **(D)** Diagrammatic representation of the process of germline inheritance of mitochondria through the pole cells.

*Drosophila* females have two ovaries, typically comprised of 16–21 ovarioles (Figure 2A). GSCs are located in the germarium that constitutes the apical end of each ovariole (Kirilly and Xie, 2007). The germarium is followed by a series of developing egg chambers arranged linearly with the most mature egg chamber at the distal end. Within the germarium, 2–3 GSCs reside at the anterior tip in close proximity to the niche. The niche consists of a cluster of 5–7 cap cells (CC) that remain connected to 8–10 tightly packed disc-like terminal filament (TF) cells (Figure 2A). GSCs typically undergo asymmetric self-renewing divisions, producing one daughter stem cell that remains attached to the cap cell and a second daughter cell displaced from the niche. The daughter cell leaving the niche, generally termed as the cystoblast (CB), undergoes four rounds of amplifying division without cytokinesis to generate a 16-cell interconnected cyst (Greenspan et al., 2015).

Apart from the GSCs, the germarium harbors two types of somatic stem cells. The ESCs divide to generate escort cells (EC) that wrap around the cystoblast and encase the dividing cysts until they reach the stage of 16 cells (Figure 2A; Margolis and Spradling, 1995). After that, the cysts get surrounded by the somatic follicle epithelial cells (FC), and they bud off the germarium as individual egg chambers. About 2–3 FSCs located at the junction between regions 2a and 2b of the germarium divide asymmetrically to give rise to the follicle epithelial cells (Figure 2A; Margolis and Spradling, 1995). Eventually, the egg chamber develops into a mature egg chamber consisting of 15 nurse cells and an oocyte.

It has been observed that while the young GSCs are characterized by the presence of relatively elongated mitochondria forming a big cluster near the fusome, with aging, fragmented mitochondria are observed in the GSCs with a concomitant increase in the expression of the mitochondrial fission gene, *drp1* (Amartuvshin et al., 2020). Inducing mitochondrial fission in young GSCs (in GSCs mutant for *dmarf*) mimics the condition observed in aged GSCs (Figure 2B). Interestingly, these GSCs with fragmented mitochondria, proliferate slowly, are low in BMP signaling, and demonstrate a tendency to move away from the niche and differentiate. Metabolically, these GSCs are characterized by low mitochondrial membrane potential and increased lipid accumulation due to defective fatty acid metabolism. In contrast, preventing mitochondrial fission (in GSCs mutant for *drp1*), as such, does not affect GSC division or maintenance. However, it seems that the *drp1* mutant GSCs exhibit increased niche occupancy (Figure 2B), at least in part, due to increased E-cadherin expression. Preventing mitochondrial fission in aged GSCs enhances their maintenance by preventing their loss due to low mitochondrial membrane potential, decreased ROS levels, and reduced BMP signaling (Amartuvshin et al., 2020). Furthermore, it has been documented that proliferative aging of the GSCs causes dramatic decrease in cytochrome C oxidase activity, impaired mtDNA replication, and accumulation of mutations on mtDNA (Ren et al., 2017). Based on these strong correlations, mitochondrial fission can be considered as an important contributing factor for age-dependent decline in GSC activity, eventually responsible for the reduced hatching rate of embryos produced by old mothers.

Gene expression profiling studies revealed that the genes associated with glycolysis and Kreb’s cycle are expressed at very low levels in the young GSCs (Figure 2B; Teixeira et al., 2015). Notably, the transcript levels of the glycolytic enzyme Aldolase and that of the Kreb’s cycle enzyme Oxoglutarate dehydrogenase are hardly detectible, implying that the GSCs acquire ATP from sources other than internal oxidative metabolism. That the GSCs do not rely on Oxphos has been further evidenced by the observation that GSC specification and maintenance remain unaffected even after knocking down each of the 13 nuclear-encoded ATP synthase subunits. However, differentiation of the germ cells gets arrested upon knocking down ATP synthase subunits (Figure 2B). Impairing the processes of mitochondrial transcription, translation, and protein import machinery, associated with expression, assembly, and oligomerization of the ATP synthase, also exhibit similar differentiation defects as the cysts cannot move from four to eight-cell stage. Interestingly, the differentiation process of female GSCs is not dependent on the ATP synthesizing capacity of this Complex, as knockdown of other members of the Oxphos pathway does not disrupt differentiation. Instead, the ATP synthase dimerization-dependent mitochondrial crista maturation serves as the critical factor for cyst maturation and subsequent differentiation (Teixeira et al., 2015). Thus, ATP synthase is not essential for stem cell maintenance or the initiation of differentiation but instead plays a crucial role in cyst differentiation independent of Oxphos.

Given the fact that mitochondria are primarily maternally inherited and mtDNA replication does not initiate in early embryonic development, oocytes are furnished with large amounts of mitochondria to cater the energy demands of early embryogenesis (Tourmente et al., 1990). However, while furnishing the oocytes with a sufficient number of mitochondria, oogenesis also limits the transmission of defective mitochondria with mtDNA mutations. Therefore, quality control of the mitochondria (Figure 2C) is of pertinent importance as compared to the nuclear genome, mitochondria have high mutation rates, low recombination levels of mtDNA, and lack of an effective repair mechanism. In order to restrain the propagation of deleterious mitochondrial mutations in subsequent generations, female germline has a robust selection mechanism that is not observed in male germline or somatic tissues, including the ovary’s soma. At the 2–8 celled cyst stage, when mtDNA replication does not occur, mitochondrial fragmentation serves as an effective selection mechanism to drive out defective mitochondria (Lieber et al., 2019). Notably, the fragmented mitochondria that adopt a rounder morphology produce low ATP, which marks these mitochondria for removal by recruiting mitophagy proteins Atg1 and BNIP3. Interestingly, genetic means of mitochondrial fragmentation by upregulating pro-fission gene *Drp-1* or downregulation of *dMfn* in female GSCs also leads to fragmentation of defective mitochondria at 2–8 celled cyst stage followed by their elimination (Lieber et al., 2019).

Intriguingly, mtDNA fitness and mitochondrial respiration are driven by an active ETC that is intertwined in the mitochondrial selection mechanism. Both mitochondrial respiration and mtDNA replication are quiescent in GSCs and dividing cysts but get markedly upregulated in the late germarium (Figure 2C). This transition is achieved by a feed-forward Insulin-Myc axis that promotes transcriptional activation of ETC genes and mtDNA replication (Wang et al., 2019). To begin with, transient activation of Jun N-terminal kinase (JNK) in the late germarium upregulates insulin receptor (InR) that boosts insulin signaling (IIS) to regulate post-translational Myc activity through Shaggy (Sgg) and 4-EBP (Thor). In turn, Myc controls ETC biogenesis and mtDNA replication. Importantly, though the expression of the InR and the initiation of insulin-Myc signaling are triggered by transient JNK signaling, Myc maintains IIS activity by boosting InR expression after JNK activity subsides. Inactivation of IIS and JNK signaling does not impact the number of eggs laid. However, it significantly reduces mtDNA’s deposition in the oocytes that negatively affects the hatching of eggs (Wang et al., 2019). Similarly, the abrogation of mtDNA replication by loss of topoisomerase IIIα (topo IIIα) leads to complete sterility. Here also, there is no defect in the egg-laying of topo IIIα mutant females, but their eggs are smaller in size with around 20-fold decrease in mtDNA content and do not hatch (Wu et al., 2010).

Apart from supplying the oocyte with adequate amounts of mtDNA, extensive mtDNA replication is essential to support another unique phenomenon termed as replication competition (Figure 2C). This refers to a process wherein wild-type mtDNA replicates and increases in mass to out-compete mtDNA carrying deleterious mutations (Zhang et al., 2019). This selective propagation of healthy mitochondria is aided by simultaneous phasing out of deleterious or damaged mitochondria. PINK1, the mitochondrial protein kinase, stabilizes on the outer membrane of the mitochondria harboring deleterious mtDNA mutations and stops the local protein synthesis by phosphorylating La-related protein (Larp), which serves as a translation stimulator. Inhibition of local protein translation on defective mitochondria limits their mtDNA replication and hence the transmission of deleterious mutations to the offspring. The absence of this mechanism in *pink1B9* mutants leads to impaired mtDNA selective inheritance (Zhang et al., 2019).

Quite interestingly, the process of germline inheritance of mitochondria also begins during oogenesis (Figure 2D). As the oocyte matures, a striking accumulation of mitochondria and mtDNA occurs toward its posterior end (Cox, 2003). This process is facilitated by the microtubule driven streaming of the oocyte cytoplasm. The long isoform of the protein Oskar alters the actin cytoskeleton to trap the mitochondria at the posterior end (Hurd et al., 2016). Post-fertilization, the first cellularization event happens in the posterior end of the syncytial embryo with pole cell formation. Therefore, these pole cells inherit the posterior end’s oocyte cytoplasm, generally termed as the germplasm, a mixture of proteins, RNAs, and mitochondria that are deposited there during oogenesis. Later during development, the pole cells give rise to the Primordial Germ Cells (PGCs), the precursors for the GSCs of the progeny flies. Thus, this fascinating mechanism ensures the germline inheritance of mitochondria from the mother to the progeny’s GSCs (Hurd et al., 2016).

Besides the GSCs, proper functioning of the FSCs also depends on mitochondrial activity. An unbiased genetic screen revealed that mitochondrial dysfunction and subsequent rise in ROS levels lead to loss of FSCs (Wang et al., 2012). While the loss in FSC by excessive accumulation of ROS is mediated by the activation of JNK pathway in a subset of the mitochondrial mutants isolated in the screen, several other mutations affecting mitochondria also lead to loss of FSC by pathways unrelated to ROS production, that remain to be identified.

## Intestinal Stem Cells of the Midgut and Their Regulation by Mitochondria

One of the rapidly turned over tissues in most animals is the intestinal epithelium, wherein cells are lost continuously from the surface. They are replenished by the proliferation of the resident ISCs (Casali and Batlle, 2009). Since their discovery, *Drosophila’s* ISCs have come up as an excellent model for adult stem cell biology (Micchelli and Perrimon, 2005; Ohlstein and Spradling, 2005; Takashima et al., 2008). They bear significant resemblance to their mammalian counterparts in terms of their modes of fate specification and their ability to respond to damage. Most of the signaling pathways involved in regulating mammalian epithelial stem cells have been evidenced to regulate the *Drosophila* ISCs (Casali and Batlle, 2009).

**Figure 3:**
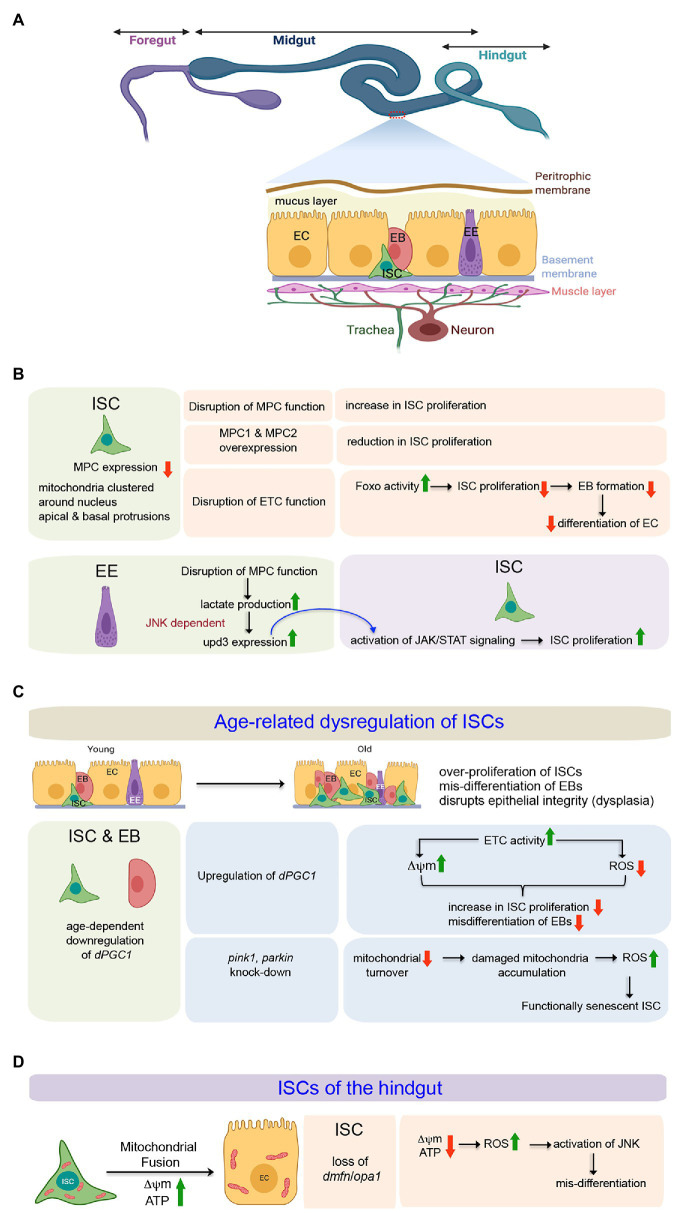
Involvement of mitochondria in defining the state and differentiation of Intestinal Stem Cells (ISCs) in the midgut and the hindgut of *Drosophila*. **(A)** Schematic representation of the adult gut of *Drosophila* highlighting the relative positions of the ISCs and the differentiating cell types in the midgut epithelium. The expanded forms of the acronyms used are provided in the text. **(B)** Table showing the importance of mitochondrial function to regulate proliferation and differentiation of midgut ISCs both in cell autonomous and non-autonomous manner. **(C)** Involvement of mitochondrial processes (biogenesis and turnover) during age-related dysregulation of ISCs in the midgut. **(D)** Mitochondrial fusion and its importance in the differentiation of hindgut ISCs.

In adult flies, the ISCs are housed in the epithelial layer of the midgut, the functional equivalent of the mammalian intestine. Structurally, the *Drosophila* midgut is comprised of a single layer of epithelium consisting of two differentiated cell types – the absorptive enterocytes (ECs) characterized by a polyploid nucleus and the relatively smaller hormone-producing enteroendocrine (EE) cells (Figure 3A). The ISCs lying adjacent to the basement membrane remain interspersed in between the ECs and EE cells (Figure 3A) along the entire length of the midgut (Miguel-Aliaga et al., 2018). A complex niche that constitutes the neighboring differentiated midgut epithelial cells, the surrounding visceral muscles, and the tracheal cells govern self-renewal, proliferation, and differentiation of the ISCs (Jin et al., 2017). The ISC of the posterior midgut usually undergoes an asymmetric division to generate a new ISC and an intermediate progenitor, enteroblast (EB; Goulas et al., 2012). Interestingly, though at a lower frequency, the ISCs can undergo symmetric division to produce either two ISCs or two EBs. In its turn, the EB can differentiate into either an EC or into an EE cell. However, recent data support that the ISCs can also directly differentiate into EE cells (Biteau and Jasper, 2014).

The ISCs, EBs, and the ECs contain mitochondria clustered around their nuclei and packed, rather than randomly distributed, in their apical and basal protrusions (Figure 3B; Endow et al., 2019). The larger protrusions of the ISCs and ECs usually have larger numbers of clustered mitochondria. Despite having many mitochondria, the ISCs primarily rely on the glycolytic metabolic program to drive their robust proliferation (Schell et al., 2017). The possible reason behind the uncoupling of glycolysis from Oxphos is the low level of expression of the genes coding for Mitochondrial Pyruvate Carrier (MPC) in the ISCs. The MPC proteins, MPC1 and MPC2, form a complex on the mitochondrion’s inner membrane that is necessary and sufficient for efficient import of cytoplasmic pyruvate into the mitochondrial matrix (Bricker et al., 2012; Herzig et al., 2012). Within the matrix, the pyruvate dehydrogenase complex (PDH) converts pyruvate to acetyl-CoA that enters the TCA cycle. Thus, MPC serves as the bridge between cytoplasmic glycolysis and mitochondrial Oxphos. Disruption of MPC function, specifically in the ISCs, increases their rate of proliferation. Conversely, specific overexpression of MPC1 and MPC2 in ISCs or progenitors suppresses ISC proliferation (Figure 3B). Therefore, by limiting mitochondrial pyruvate flux, MPC plays a cell-autonomous role in maintaining the proliferation of ISCs (Schell et al., 2017). However, a very recent study demonstrates that mitochondrial Oxphos is essential for *Drosophila* ISC proliferation (Zhang et al., 2020). Genetic disruption of the mitochondrial ETC in the ISCs by manipulating the mitochondrial genome retards their proliferation rate, leading to the production of a limited number of EBs, which eventually fails to differentiate into ECs (Figure 3B). While RNAi mediated disruption of each ETC complex impairs ISC proliferation to some extent, a much stronger effect is observed upon knocking down members of complexes III and IV of the ETC. Notably, FOXO protein and its transcriptional activity are elevated in these ISCs and EBs without any detectable cellular ROS increase. Furthermore, knocking down *foxo* in these ISCs markedly suppresses the proliferation and lineage specification defects associated with the ETC disruption. Therefore, mitochondrial respiration is critical for *Drosophila* ISC proliferation and lineage specification and acts at least partially by repressing endogenous FOXO signaling (Zhang et al., 2020).

Quite interestingly, the mitochondrial function of the ECs also impacts the behavior of the ISCs (Figure 3B; Wisidagama and Thummel, 2019). Genetic loss of the MPC in ECs leads to increased lactate production by lactate dehydrogenase (LDH) that induces Upd3 expression in a JNK-dependent manner. The secreted cytokine Upd3 activates JAK/STAT signaling in the ISCs, promoting their proliferation. Interestingly, the increased proliferation of ISCs in response to the loss of MPC function in the EC can be suppressed by disrupting LDH function in the EC. However, under normal conditions, disruption of LDH in ECs does not impact ISC proliferation. These results imply the involvement of lactate in inducing ISC proliferation due to altered MPC function in the EC (Wisidagama and Thummel, 2019).

The *Drosophila* midgut exhibits dramatic changes as the animal ages (Figure 3C). One of the hallmark features is epithelial dysplasia, characterized by ISC overproliferation and aberrant differentiation of EBs (Rodriguez-Fernandez et al., 2020). Both cell-extrinsic and cell-intrinsic factors contribute to the continuous proliferative activation of ISCs. The cell-extrinsic factors include activation of JNK and PDGF/VEGF signaling by chronic production of ROS due to a shift in the composition of the gut microbiota (Buchon et al., 2009). A variety of intrinsic signaling factors that include a decline in mitochondrial function (Rera et al., 2011), drop in Nrf2 activity (Hochmuth et al., 2011), increased endoplasmic reticulum stress (Wang et al., 2014), as well as changes in autophagy (Rodriguez-Fernandez et al., 2019), contribute toward the disruption of ISC homeostasis. The overproliferating ISCs give rise to EBs that initiate but fail to differentiate into ECs and accumulate on the epithelium’s basal side, disrupting its structure and gut’s function. This age-related dysregulation is associated with a gradual decline in the expression of *dPGC-1/spargel*, which codes for the peroxisome proliferator-activated receptor *γ* coactivator 1, a central regulator of energy metabolism and mitochondrial biogenesis (Rera et al., 2011). Overexpression of *dPGC-1*, specifically in the ISCs and the EBs results in a delay of age-related dysregulation of the midgut (Figure 3C). The underlying mechanism involves an increase in the Complex I and Complex II activities of the mitochondrial ETC, which not only prevents the progressive loss of mitochondrial membrane potential but also lowers the ROS levels throughout the intestinal epithelium. Maintenance of mitochondrial activity and lowering ROS levels, in turn, abrogates the dramatic increase in ISC proliferation and accumulation of misdifferentiated daughter cells in the midgut that generally occurs during aging (Rera et al., 2011). Importantly, in the absence of transit-amplifying daughter cells due to direct differentiation of EBs into either of the two lineages, the effects of manipulating *dPGC-1* in ISCs/EBs are not only restricted to the stem cells but are also reflected throughout the midgut. Nonetheless, it is evident from these studies that proper mitochondrial activity blocks age-dependint epithelial dysplasia of the gut by inhibiting the ISCs from undergoing overproliferation.

Interestingly, age-dependent downregulation of dPGC-1 in the midgut is associated with an age-dependent increase in the expression of the gene *Indy* (*I’m not dead yet*) that codes for a plasma membrane transporter of the Kreb’s cycle intermediate, citrate (Rogers and Rogina, 2014). The reduction of INDY activity, either by genetic means or by calorie restriction, leads to an increase in the midgut expression of *dPGC-1* accompanied by enhanced mitochondrial biogenesis and reduction in ROS levels. These physiological changes prevent the ISCs from undergoing excessive proliferation and thereby preserve ISC homeostasis. Thus, by modulating *dPGC-1*, INDY functions as a physiological regulator of the mitochondrial function of the ISCs in response to changes in nutrient availability and organismal needs (Rogers and Rogina, 2014).

A remarkable improvement in age-dependent dysregulation of gut homeostasis has also been reported upon RNAi-mediated knockdown of the mitophagy related genes, *pink1* and *parkin* in the ISCs/EBs (Figure 3C; Jones et al., 2017). ISC/EB–specific reduction of Pink1 and Parkin blocks mitochondrial turnover and eventually leads to the accumulation of damaged mitochondria with swollen or collapsed morphology and high ROS levels. Interestingly, despite having elevated ROS levels, the ISCs do not undergo uncontrolled proliferation, a hallmark feature for age-related changes in the midgut. Instead, the ISCs, with accumulated damaged mitochondria within them, become functionally senescent, thereby highlighting that: (a) ISCs use mitophagy as one strategy to maintain a healthy complement of mitochondria and (b) there exists a mechanism by which ISC/EB–specific mitochondrial dysfunction uncouples cellular and tissue aging to maintain the organization of the intestinal epithelium (Jones et al., 2017). Even though elevated levels of ROS upon downregulation of *pink1* and *parkin* prevent excessive proliferation of the ISCs, increased ISC proliferation is generally observed upon treatment with the ROS-inducing compound Paraquat (Biteau et al., 2008) as well as in mutants for the ROS scavenging enzyme catalase (Choi et al., 2008). Conversely, treating flies with N-Acetyl-Cysteine and Glutathione limit ISC proliferation (Buchon et al., 2009). Analogous to female GSCs, the Keap1 and Nrf2 redox management system establishes a switch that controls the proliferation of ISCs (Hochmuth et al., 2011). In young flies, under homeostatic conditions, constitutively active Nrf2 induces antioxidant genes, such as *gstD1*, *gclc*, and *jafrac1*, to maintain low intracellular ROS levels and thereby prevents excessive proliferation. In contrast, in aged flies and during mitogenic and stress conditions, Keap1 mediated inhibition of Nrf2 prevents the antioxidant genes’ expression. As a result, the intracellular level of ROS increases, allowing the ISCs to proliferate (Hochmuth et al., 2011). Therefore, Keap1 and Nrf2 seem to control a shift from a resting, largely quiescent state of ISCs to a condition of active proliferation.

## Mitochondrial Regulation of the ISCs of the Hindgut

Morphologically, the hindgut of adult *Drosophila* can be divided into four distinct regions. In the anterior-most part, adjacent to the midgut is the hindgut proliferation zone (HPZ). This is followed by the pylorus, the ileum, and the rectum. It has been documented that the HPZ contains a narrow band of ISCs that proliferates and differentiates into enterocytes to repair the pylorus in response to injury (Takashima et al., 2008; Fox and Spradling, 2009). While these ISCs have very few mitochondria that are round, small, and devoid of cristae, the differentiated adult hindgut enterocytes are characterized by densely packed mitochondria that are highly branched (Figure 3D; Deng et al., 2018). Impairing mitochondrial fusion by knocking-down of *opa-1* or *marf* in the ISCs blocks EC differentiation without affecting their proliferation. However, blocking mitochondrial fission either by over-expression of *marf* or downregulating *drp1* does not lead to differentiation defects. Importantly, the block in differentiation, as observed upon knocking down *opa1*, can be rescued by concomitant inhibition of *drp-1*. One possible reason for the differentiation defect of the hindgut ISCs knocked down for *opa1* is ectopic induction of JNK signaling by elevated ROS levels as scavenging ROS leads to partial rescue of *opa1RNAi*-associated differentiation defects by downregulating JNK activity (Deng et al., 2018). Thus, the outcome of this study demonstrates that the self-renewal of the ISCs in the hindgut is mostly independent of mitochondrial function, and a progressive fusion process is essential for EC differentiation.

## Role of Mitochondria in Neural Stem Cells

**Figure 4:**
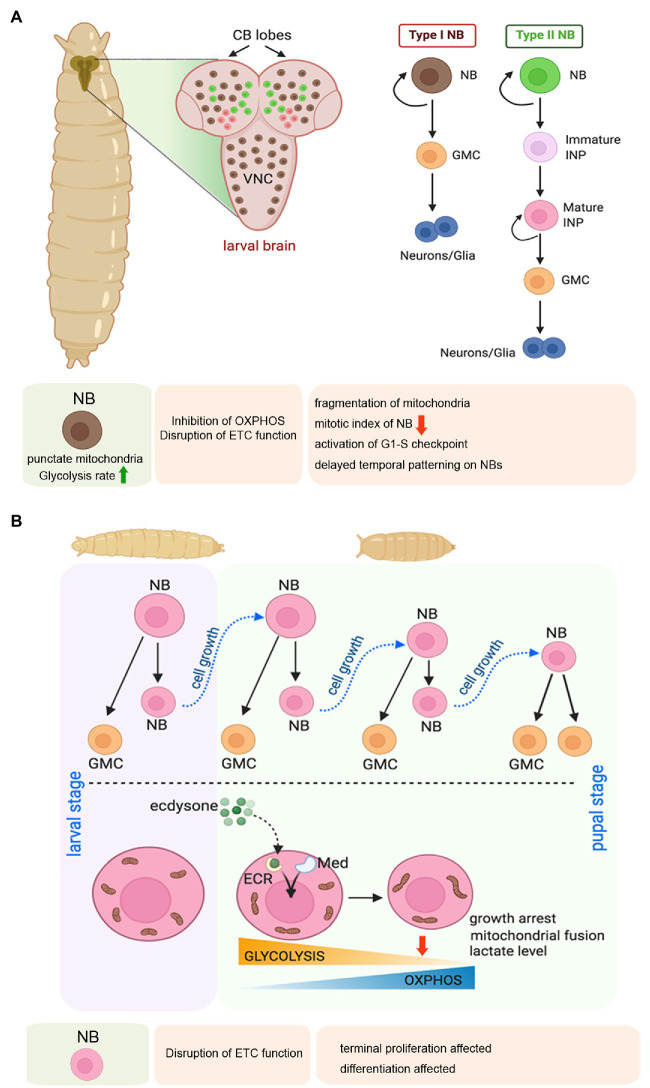
Mitochondrial regulation of larval neuroblast (NB) proliferation, and terminal differentiation of NBs during early pupal stage in *Drosophila*. **(A)** Schematic representation of the larval brain showing the relative position of Type 1 and Type 2 NBs, and their pattern of self-renewal and differentiation. The expanded forms of the acronyms used are provided in the text. Table showing the effects of disrupting mitochondrial function in the larval NBs. **(B)** Schematic representation of the changes in mitochondrial morphology and function associated with NB growth arrest and terminal differentiation at the larval to pupal transition.

Adult *Drosophila* usually does not have NSCs. NSCs are present in the embryonic and larval stages, and at the onset of pupariation, they undergo growth restriction accompanied by terminal differentiation or apoptosis (Homem and Knoblich, 2012). *Drosophila* NSCs are also known as neuroblasts (NBs). During the embryonic stage, after their formation, the NBs delaminate from the neuro-epithelium and divide asymmetrically to give rise to another NB (self-renewal) and a ganglion mother cell (GMC; Hartenstein et al., 1987). Eventually, the GMC further divides and differentiates into a pair of neurons or glia. By late embryogenesis significant population of the NBs become quiescent, only to re-enter into active cell division in the larval stages (Sousa-Nunes et al., 2011). In the larvae, NBs are primarily found in the central brain and in the ventral nerve cord (Figure 4A). The central brain consists of three types of NBs, namely type I, type II, and mushroom body neuroblasts. Type I and Type II NBs differ from each other based on their division profile. While a Type I NB undergoes stereotypic division to give rise to one NB and one GMC that further divides to produce two neurons or glia, a Type II NB divides to generate one NB and another transit-amplifying cell, termed as the intermediate neural precursor (INP). The INP undergoes maturation, following which the matured INP divides asymmetrically to produce one GMC, and another matured INP. Therefore, repeated division of the matured INP gives rise to a pool of GMCs that differentiate into neurons or glia (Figure 4A; Reichert, 2011; Harding and White, 2018).

One of the fascinating aspects of the *Drosophila* NSCs is their entry into a quiescent stage during late embryogenesis. These quiescent NSCs (qNSCs) again start dividing during early larval stages with the initiation of active feeding (Truman and Bate, 1988; Reichert, 2011). It has been observed that the qNSCs are characterized by a cytoplasmic protrusion (Endow et al., 2019), typically not observed in dividing NSCs. The cytoplasmic protrusions connect the qNSCs to the brain region known as neuropil. These cytoplasmic protrusions of qNSC are enriched with clustered mitochondria and have been proposed to be essential for maintaining the stemness in the quiescent stage. The protrusions have been proposed to connect qNSCs and its niche by forming the stem-cell-to-niche mitochondrial bridges that could sense niche signals (Endow et al., 2019). However, a detailed analysis in this intriguing finding is necessary to dissect the exact role of these cytoplasmic protrusions in qNSCs.

In larval stages, the proliferating NBs are characterized by punctate mitochondria and an increased rate of glycolysis (Figure 4A; Homem et al., 2014). Therefore, the NBs are thought to support their growth and proliferation by generating ATP through aerobic glycolysis rather than Oxphos. According to this school of thought, mitochondrial respiration is dispensable for the proliferating larval *Drosophila* NSCs. However, another study has evidenced that mitochondrial Oxphos is an essential contributor to the proliferation of the NBs and generates diversity through temporal patterning (van den Ameele and Brand, 2019). According to them, inhibition of Oxphos in the NBs throughout development results in the formation of smaller brains. In contrast, inhibition of glycolysis by NB-specific knockdown of phosphofructokinase (PFK), Aldolase, or phosphoglycerate kinase (PGK) does not affect brain size. Extending the thought further, they demonstrate that NB specific knockdown of Complex I and Complex V of the ETC causes mitochondrial fragmentation leading to a marked reduction of mitotic index in NBs of larval third instar VNC. Furthermore, attenuating Oxphos causes activation of the G1/S checkpoint. In turn, this delays temporal patterning of larval NSCs, implying that mitochondrial function is required for NSCs to progress from an early to a late temporal fate. Temporal patterning refers to a gradual process of differential gene expression that allows the NBs to generate progenies with diverse identities according to their developmental time (van den Ameele and Brand, 2019). Thus, temporal patterning helps in generating the diversity of neurons and glia within the CNS. Therefore, mitochondrial respiration is critical for NB proliferation and subsequent differentiation during the larval stage.

Survival and differentiation of the larval NBs also rely on their mitochondrial calcium homeostasis (Lee et al., 2016). It has been demonstrated that Miro, apart from its role in mitochondrial transport machinery, is required for NB maintenance mainly by regulating mitochondrial calcium homeostasis. Mechanistically, Polo Kinase-mediated phosphorylation promotes the localization and interaction of Miro with the calcium transporters at the ER-mitochondria contact site (ERMCS). Inactivation and overexpression of Miro, both impair the maintenance and lineage development of the NB; although through different mechanisms. While loss of Miro activity causes mitochondrial calcium depletion, and metabolic impairment leading to premature differentiation and loss of NB through a non-apoptotic mechanism, overexpression of Miro induces apoptotic response by causing mitochondrial calcium overload, oxidative stress, and activation of the apoptotic cascade. Beyond its role in NB survival, proliferation, and differentiation during larval development, a recent study has implicated the importance of mitochondria in immortalization of larval NBs during tumorigenesis (Bonnay et al., 2020). Employing single-cell transcriptomics, targeted metabolomics, and *in vivo* genetic screening, it has been demonstrated that extensive mitochondrial fusion mediated metabolic reprogramming is the rate-limiting step for immortalization of stem cells during tumorigenesis. The metabolic reprogramming includes a metabolic switch from glycolysis to Oxphos, and increased NAD^+^ biogenesis. Direct inhibition of Oxphos or that of mitochondrial fusion halts these transformed NBs in quiescence and prevents tumorigenesis.

The transition from larval to pupal phase is of prime importance for the *Drosophila* NBs (Figure 4B). In this period, the proliferating larval NBs enter a phase of growth restriction as they stop dividing within the first 20–30 h of pupation and differentiate or undergo apoptosis (Truman and Bate, 1988). This cell cycle exit of early pupal central brain NBs is mediated by a metabolic switch from glycolysis to Oxphos induced by the Ecdysone hormone and Mediator complex (Homem et al., 2014). This transition is associated with a dramatic shift in mitochondrial morphology. While the mitochondria of larval and early pupal brains show a punctate morphology, 8–10 h after pupa formation, the mitochondrial morphology changes to a more fused form, and making a reticular network. This change is accompanied by a marked drop in lactate levels, indicating a switch from glycolysis to Oxphos. Two independent studies (Homem et al., 2014; van den Ameele and Brand, 2019) have demonstrated that depletion of subunits of Complex I, III, IV, or V of the mitochondrial ETC in NSCs prevents their termination of proliferation and timely differentiation at the onset of pupal life. However, a difference exists in their interpretation of the underlying mechanism. Whereas one group suggests that Oxphos depletes metabolites for biosynthesis and reduces growth and induces NBs to stop proliferating in pupae (Homem et al., 2014), the other group argues that the termination of NB proliferation is a consequence of the temporal patterning defects caused by Oxphos dysfunction (van den Ameele and Brand, 2019). Nonetheless, both studies implicate the importance of Oxphos in cell cycle exit and terminal differentiation of the *Drosophila* NSCs.

## Role of Mitochondria in Hemocyte Precursors

**Figure 5:**
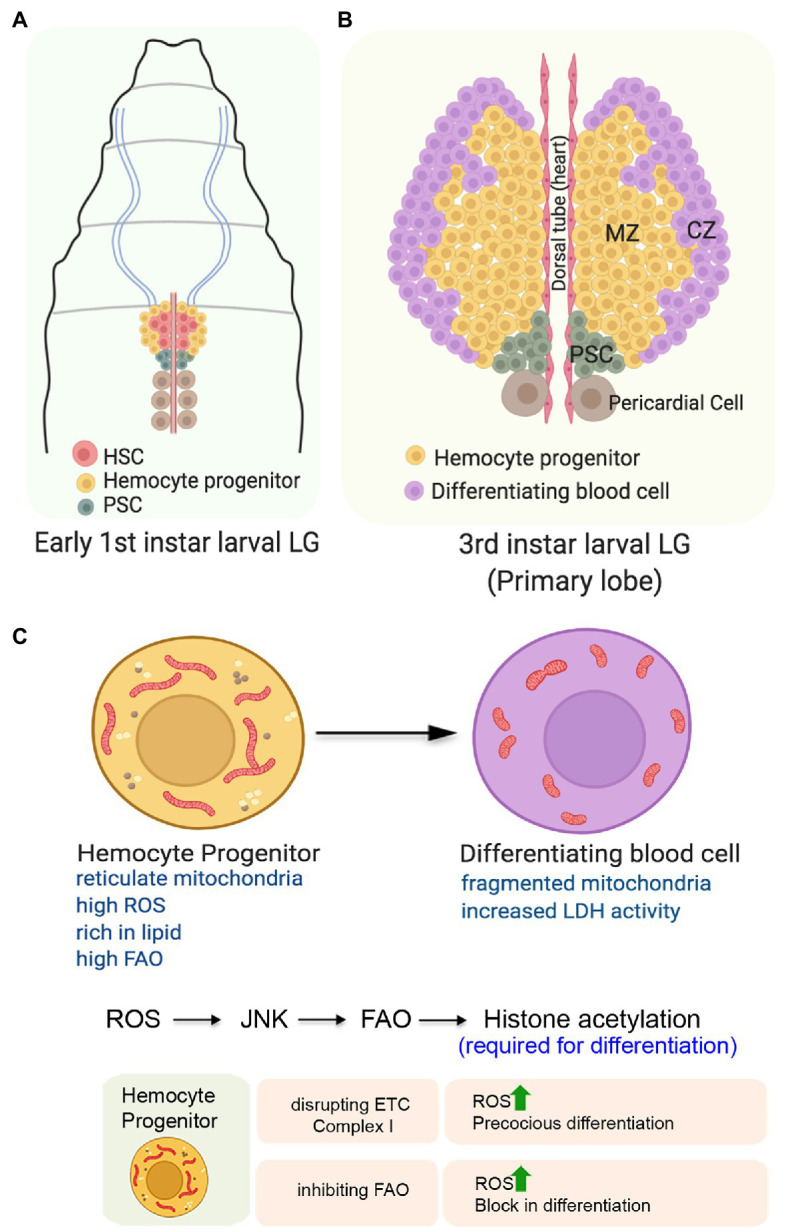
Role of mitochondria in determining the state and fate of hemocyte progenitors in the larval lymph gland of *Drosophila*. **(A)** Organization of the early first instar larval lymph gland showing the Hematopoietic Stem Cells (HSCs), the hemocyte progenitors, and the niche cells. **(B)** Schematic representation of the third instar larval lymph gland depicting the position of the different zones. The expanded forms of the acronyms used are provided in the text. **(C)** Changes in mitochondrial form and function and its importance for the differentiation of the hemocyte progenitors. Table showing the outcome of altering mitochondrial function in the hemocyte progenitors.

The process of hematopoiesis occurs in two waves in *Drosophila*. The first wave of hematopoiesis, referred to as the primitive hematopoiesis, occurs in the embryonic head mesoderm, giving rise to both circulating and sessile population of blood cells present throughout the life span of the flies (Tepass et al., 1994). The second wave of hematopoiesis or the definitive hematopoiesis occurs in a defined multi-lobed larval hematopoietic organ, the lymph gland (LG; Jung et al., 2005), which develops from the cardiogenic mesoderm during the embryonic period (Mandal et al., 2004). The founder cells of the lymph gland show several resemblances to the Aorta-Gonad-Mesonephros (AGM) HSCs (Figure 5A; Mandal et al., 2004; Dey et al., 2016). For their transient presence in the embryonic and first instar larval stages, these HSCs depend on the Decapentaplegic (Dpp) signal from the hematopoietic niche. Eventually, the HSCs divide and give rise to stem-like hemocyte progenitors (Dey et al., 2016). The role of mitochondria or metabolism in this transient population of HSCs is yet to be demonstrated.

The first or the primary lobe of the third instar larval lymph gland consists of three zones (Figure 5B). The transit amplifying stem-like hemocyte progenitors present in the central region constitutes the medullary zone (MZ). These multipotent progenitor cells can give rise to all blood cell lineages that populate the gland’s outer periphery, referred to as the cortical zone (CZ). A group of 40–50 cells, located posterior to both of these zones forms the Posterior Signaling Center (PSC) that serves as the niche. Through an intricate regulatory network, the PSC maintains the homeostasis between the progenitors and the differentiating hemocytes (Evans et al., 2003; Banerjee et al., 2019).

The hemocyte progenitors in the primary lobes of the late third instar larval lymph gland have a fused and extensive reticular network of mitochondria (Figure 5C). Compared to the progenitors, the differentiating hemocytes with less reticular mitochondria, higher glucose uptake potential, and increased LDH enzymatic activity demonstrate their high glycolytic dependence (Tiwari et al., 2020). Though not established, this unexpected glycolytic dependence of the differentiating hemocytes might be because of their proliferative state. These differentiating hemocytes which demonstrate increased rate of cell division may rely more on glycolysis, rather than Oxphos. Together, these results suggest a metabolic dichotomy wherein differentition of the hemocyte precursors is associated with a shift from mitochondrial Oxphos to glycolysis.

Intriguingly, under normal physiological conditions, the hemocyte progenitors are characterized by elevated levels of ROS (Owusu-Ansah and Banerjee, 2009). It is believed that the increased levels of ROS actually primes these progenitors for differentiation as genetically scavenging ROS by overexpressing antioxidant protein Gtpx1 leads to a block in differentiation. Conversely, a further increase in ROS levels either in SOD2 hypomorphic mutants or by targeted knockdown of ETC Complex I subunits ND75 and ND42 leads to precocious differentiation of the hemocyte progenitors (Figure 5B). ROS mediated differentiation of the hemocytes is achieved by downregulation of the Polycomb group of genes in a JNK and FOXO dependent manner (Owusu-Ansah and Banerjee, 2009). In an independent study, it has been demonstrated that mitochondrial ROS promotes differentiation of hemocyte progenitors by reducing the levels of E-cadherin (Gao et al., 2014). Knocking down of the anti-oxidant genes, Sod2 and Catalase lead to a reduction in the levels of Shotgun protein (the fly homolog of E-cadherin) generally expressed at a relatively higher level in the MZ. Temporally, the drop in Shotgun levels precedes the loss of Odd-skipped-expressing hemocyte precursors. In contrary, over-expression of Shotgun prevents the hemocyte progenitor differentiation even under conditions of paraquat-induced oxidative stress.

A very recent study has provided an unexpected directionality in our understanding of the importance of mitochondrial function in the hemocyte progenitors (Tiwari et al., 2020). This study demonstrates that in the primary lobe of the third instar larval lymph glands, the hemocyte progenitors arrested in the G2/M phase of the cell cycle are rich in lipid content and express various FAO enzymes and Hnf4; the major transcription factor implicated in larval fat mobilization and FAO. Inhibiting mitochondrial FAO by genetic or pharmacological means leads to a block in the differentiation of hemocyte progenitors despite having high ROS levels in them (Figure 5B). Besides impeding differentiation, inhibiting FAO also disrupts the G2/M arrest. On the other hand, the upregulation of FAO in hemocyte progenitors leads to precocious G2/M arrest and differentiation. The mechanistic basis of FAO dependent hemocyte progenitor differentiation involves the requirement of Acetyl-CoA, the end product of FAO, for histone acetylation necessary for differentiation. Their genetic and molecular analyses reveal that FAO acts downstream to the ROS-JNK axis, as the expression of CPT1/whd (*withered*), the rate-limiting enzyme of FAO, is transcriptionally regulated by JNK (Tiwari et al., 2020). These findings provide an elegant connection between cellular signaling machinery and mitochondrial FAO to promote differentiation of the stem-like hemocyte progenitors in *Drosophila*.

Apart from the hemocyte progenitors, the niche cells present in the PSC that maintains blood progenitors can regulate the cellular immune response by sensing oxidative stress (Sinenko et al., 2011). While under normal developmental conditions, the PSC cells are characterized by low levels of ROS, attenuating mitochondrial function by knocking down ND75 causes a readily detectable increase in ROS levels. The phenotypic consequences of generating oxidative stress in the PSC cells is a significantly robust increase in the numbers of both circulating and lymph gland-resident lamellocytes, the specialized cells involved in innate immune responses. Most intriguingly, pathogen infection also induces ROS in the niche cells, resulting in the secretion of an epidermal growth factor-like cytokine signal that leads to the transdifferentiation of the circulating plasmatocytes into lamellocytes and the differentiation of hemocyte progenitors to lamellocytes.

## Commonalities and Uniqueness

Lessons learned from *Drosophila* have provided valuable insights into our understanding of mitochondrial regulation of stem cell functions. Despite tissue-specific variations, identification of some processes portrays a typical unifying role of mitochondria in governing the stem cell state and fate. Most importantly, the unifying properties are not only restricted to different types of adult stem cells in flies, but are also apparent in a vast majority of mammalian adult stem cells. This remarkable nature of resemblance highlights the evolutionary conserved role of mitochondria in stem cell maintenance and renewal.

Akin to murine and human adult stem cells that include bone marrow derived mesenchymal stem cells (Chen et al., 2008), NSCs (Wang et al., 2010; Lange et al., 2016), and hematopoietic stem cells (Simsek et al., 2010; Miharada et al., 2011; Takubo et al., 2013; Yu et al., 2013), the ISCs and NSCs in flies are characterized by the presence of punctate mitochondria and are highly dependent on glycolysis for their maintenance and/or self-renewal. Interestingly, despite having reticulate mitochondrial network, most probably, the fly GSCs also does not rely on OXPHOS for their maintenance. Impairing ATP synthase activity bears no consequence on GSC state. Rather, the results indicate that the GSCs, in addition to glycolysis, rely on mitochondrial FAO for their maintenance. Genetic perturbation that inhibits mitochondrial fusion causes increased lipid accumulation due to defective mitochondrial FAO in both male and female GSCs. This leads to loss of stemness as they undergo precocious differentiation. In fact, the dependence of adult stem cells on mitochondrial FAO and lipid metabolism for their maintenance has also been evidenced in mammalian HSCs (Ito et al., 2012) and NSCs (Knobloch et al., 2013). Related to this, even different types of tumor-initiating stem cells or cancer stem cells (CSCs) utilize mitochondrial FAO for self-renewal and resistance to chemotherapy (Samudio et al., 2010; Chen et al., 2016). Alterations in lipid metabolism not only satisfy the energy demands and biomass production of CSCs but also play an important role in the activation of several important oncogenic signaling pathways and redox homeostasis (Carracedo et al., 2013; Park et al., 2016; Noto et al., 2017). In this context, it is important to note that, similar to the GSCs, the *Drosophila* hemocyte precursors of the developing larval lymph gland also harbor elongated and reticulate mitochondria and exhibit elevated levels of mitochondrial FAO. However, unlike that observed for GSCs, disrupting mitochondrial FAO impedes the differentiation of the precursors. In terms of changes in mitochondrial morphology associated with differentiation, the hemocyte precursors also stand out from other stem cells. While differentiation of the stem cells, in most instances, is associated with the gradual fusion of mitochondria forming a reticular network, the differentiating hemocytes in *Drosophila* have isolated and less reticulate mitochondria compared to their precursors.

Mitophagy refers to the process of selective degradation of deleterious/damaged mitochondria by autophagy and thus, holds a pivotal position for mitochondrial quality control in a cell. Accumulating evidence suggests that mitophagy related processes are vital for stem cell maintenance and homeostasis. Mitophagy mediated regulation of stem cell function has been very well documented in *Drosophila* female germline. Since the offsprings inherit most of their mitochondria from their mother and mitochondrial biogenesis does not initiate early during embryonic development, the quality and the number of mitochondria contributed by the mother in the egg is of prime importance. To achieve this, a robust system of mitochondrial quality control exists in the female germarium, wherein the fragmented mitochondria with low ATP signatures are earmarked for degradation and removed by the recruitment of mitophagy components Atg1 and BNIP3. Mitophagy in ISCs has also been implicated in maintaining the healthy complement of mitochondria and age-related downregulation of mitophagy components *Pink1/Parkin* causes accumulation of damaged mitochondria in ISCs-EBs, which alters their redox state and leads to their senescence.

Studies in the recent past have implicated mitochondrial ROS as an important factor to regulate stem cell activity. In adult stem cells, this role changes in a context dependent manner. For instance, osteogenic induction of human bone marrow-derived MSCs is associated with a reduction in the ROS level (Chen et al., 2008); whereas ROS generated by Complex III of the mitochondrial ETC is required to initiate adipocyte differentiation in primary human MSCs (Tormos et al., 2011). In flies also the effects of altered ROS levels vary from one adult stem cell type to another. While elevated ROS levels in male GSCs as well as in the hemocyte progenitors of the larval lymph gland lead to their precocious differentiation, increased ROS levels block differentiation of hindgut ISCs. Age-dependent impact of ROS on stem cell fate is also evidenced in the ISCs. Overproliferation and misdifferentiation of ISCs with a concomitant increase in the number of EBs, leading to epithelial dysplasia are an outcome of increased ROS.

## Conclusion

In this review, we have put together the *in vivo* evidence that establishes how mitochondria influence stem cell function across a range of different tissue contexts in *Drosophila*. Besides highlighting the importance of mitochondrial morphology and dynamics in ascertaining the stemness and differentiation potential of a diverse array of stem cells, the outcome of these studies shed light on the changes in cellular redox state triggered by mitochondrial ROS responsible for impacting stem cell proliferation, differentiation, and senescence. Furthermore, these studies provide compelling evidence for a robust mechanism that ensures mitochondrial quality control, specifically during the onset of GSC differentiation in female flies. Despite the progress made, this research field is still in its infancy, as several issues need to be addressed. While, in some instances, the critical retrograde signaling pathways that control the process are still in the dark, in some other cases, the mechanisms that underpin the heterogeneity of mitochondrial control in different stem cell types remain undefined. Likewise, it is not evident what induces the age-dependent changes in mitochondrial structure and function that lead to stem cell aging and senescence. It is also essential to unveil the critical signaling pathways that mediate the senescence in response to mitochondrial dysfunction. Indeed, it would be intriguing to figure out whether the mechanistic basis for this process is identical in all stem cell populations or unique for each stem cell type.

With a trove of advanced genetic tools, easy accessibility of different types of stem cells, and advanced imaging techniques, *Drosophila* holds the promise to address most of these issues in the future. Application of the genetically encoded fluorescent probes, such as roGFP^mito^ (Soberanes et al., 2009) and mitoTimer (Laker et al., 2014), would also help explore the mitochondrial oxidation state and mitochondrial protein turnover/segregation in defining the stem cell state. The advent of single-cell technologies, such as single-cell RNA sequencing, combined OMICS approaches, and the use of emerging sophisticated gene-editing methods would offer an unprecedented opportunity for investigations into the endogenous heterogeneity of mitochondrial regulation of stem cell behavior. Unraveling the mechanisms in flies might help us modulate mitochondrial function as effective strategies in regenerative medicine to control stem cell proliferation, activation, and aging.

## Author Contributions

ST and SM were involved in conceptualization, design, and writing of the manuscript. SM prepared the models in collaboration with Lolitika Mandal. Both the authors contributed to the article and approved the submitted version.

### Conflict of Interest

The authors declare that the research was conducted in the absence of any commercial or financial relationships that could be construed as a potential conflict of interest.
